# Virulence and pathotype variability for *Puccinia striiformis* f. sp. *tritici* across different geographical regions and epidemic zones of China

**DOI:** 10.1186/s12870-026-08249-8

**Published:** 2026-02-05

**Authors:** Firdous Hina, Yuan Li, Qiang Yao, Bo Zhang, Faisal Shafiq Mirza, Muhammad Mubashar Zafar, Abdul Razzaq, Baotong Wang, Qiang Li

**Affiliations:** 1 State Key Laboratory of Crop Stress Resistance and High-Efficiency Production, College of Plant Protection, Northwest A&F University, Yangling, Shaanxi 712100 China; 2 Academy of Agricultural and Forestry Sciences, Qinghai University, Xining, Qinghai 810016 China; 3 Institute of Plant Protection, Gansu Academy of Agricultural Sciences, Lanzhou, Gansu 730070 China; 4 Department of Plant Breeding and Genetics, University of Agriculture Faisalabad, Faisalabad, Pakistan; 5 Institute of Molecular Biology and Biotechnology, The University of Lahore, Lahore, Pakistan

**Keywords:** Wheat stripe rust, *Puccinia striiformis* f. sp. *Tritici*, Virulence, Race characterization

## Abstract

Wheat Stripe rust, caused by *Puccinia striiformis* f. sp. *tritici* (*Pst*), poses a significant threat to wheat production globally. Understanding regional virulence patterns is crucial for effective resistance gene deployment and sustainable disease management. A total of 209 *Pst* isolates were collected from six provinces (Gansu, Qinghai, Shaanxi, Hubei, Henan, and Jiangsu) across two major epidemic regions in 2023–2024. Isolates were tested against 19 Chinese differential hosts and 18 *Yr* single-gene differentials. Using Chinese differentials, 54 races were identified, with CYR34 (19.62%), CYR32 (10.53%), and CYR33 (9.09%) being predominant; 31 were new. The *Yr* single-gene differential detected 63 races, VR1 (10.05%), VR2 (6.22%), and VR3 (5.74%) were the most frequent in the VR (Virulence Race) system, where numbers indicate sequential race designations based on distinct virulence patterns. Virulence assessment showed overwhelming prevalence against 17 Chinese varieties and 10 single-gene lines (> 60% frequency), while complete susceptibility absence occurred against Zhong 4 (*Yr* genes unknown), *Triticum spelta Album* (*Yr5*), Avocet S*6/*Yr5*, and Avocet S*6/*Yr15*. This study validates the superior discriminatory power of *Yr* single-gene differentials for *Pst* race identification and provides essential baseline data for regional resistance breeding programs. The findings support the adoption of enhanced surveillance systems for sustainable stripe rust management in China.

**Supplementary Information:**

The online version contains supplementary material available at 10.1186/s12870-026-08249-8.

## Introduction

Wheat (*Triticum aestivum* L.) is one of the world's most important cereal crops, providing approximately 21% of human caloric intake and serving as a primary protein source for billions of people globally [2, 32]. However, wheat production faces numerous biotic challenges, among which stripe rust (yellow rust), caused by *Puccinia striiformis* Westend. f. sp. *tritici* Erikss-(*Pst*) represents one of the most devastating fungal diseases globally [8, 40]. Stripe rust can cause yield losses ranging from 10–70% in susceptible cultivars under favorable environmental conditions [6, 12]. Recent studies from different global regions confirm the widespread economic impact of stripe rust. In Egypt, severe yield losses ranging from 2.72% to 69.33% were reported across different wheat cultivars during 2017–2019, with some varieties like Gemmeiza 11experincing losses exceeding 64% [30]. The pathogen's ability to undergo long-distance spore dispersal and rapid genetic recombination has resulted in the continuous emergence of new virulent races, leading to frequent breakdowns of resistance genes in commercial wheat cultivars [4, 28]. Similar patterns of aggressive race emergence and virulence evolution have been documented across diverse global regions, including significant virulence changes in Middle Eastern populations [25, 30]. China represents the world's largest stripe rust epidemic area, with the disease occurring across multiple agroecological zones [21, 33]. The country experiences regular epidemics, with major outbreaks recorded in 1950, 1964, 1983, 1990, 2002, 2017, and 2020, resulting in cumulative yield losses exceeding 13.9 million metric tons [17, 26]. The complex epidemiological system in China involves autumn over summering regions, spring multiplication areas, and epidemic zones, with pathogen populations showing distinct regional characteristics [4, 37].

Effective stripe rust management relies heavily on the deployment of resistant wheat cultivars carrying effective resistance genes [7, 22]. The importance of systematic resistance gene evaluation has been further emphasized through modern marker-assisted selection approaches, which enable more precise identification and deployment of effective resistance genes in wheat breading program [31]. However, the continuous evolution of *Pst* populations necessitates regular monitoring of race composition and virulence frequencies to guide resistance breeding programs [13, 35]. Traditional race identification in China has relied on a set of 19 Chinese differential hosts, while useful for historical continuity, may lack the resolution needed for precise virulence characterization [37]. Recent advances in stripe rust research have led to the development of a standardized set of 18 *Yr* single-gene differentials, offering several advantages over traditional differential sets, including known genetic backgrounds, single-gene specificity, and enhanced discriminatory power [10, 35]. This system has been successfully implemented in various countries, providing valuable insights into *Pst* population dynamics and facilitating international comparison of race data [36, 39]. Zhou et al. [45] conducted a comprehensive national survey using both Chinese and *Yr* single-gene differentials, demonstrating the superior resolution of the latter system and providing a baseline for national *Pst* monitoring. However, regional studies remain essential for understanding local pathogen dynamics and informing target management strategies. Regional variation in environmental conditions, cropping systems, and wheat cultivar deployment can significantly influence local *Pst* population structure [14, 42]. The objectives of the present study were to identify the races of *Pst* isolates collected in *Pst* over-summering region Gansu, Qianghai, and bridge zone region Shaanxi, winter spore production region Henan and Hubei, and spring epidemic region Jiangsu in China, and determine their distributions and frequencies using both the 19 Chinese differential and the 18 *Yr* single-gene differentials, and compare the results of the two sets of differentials. This study may set an example for using the *Yr* single-gene differentials to identify races of *Pst* in China, and the results can provide a scientific basis for monitoring *Pst* populations, guiding resistance breeding, and implementing stripe rust management based on resistance genes in wheat cultivars.

## Materials and methods

### Sample collection

**Fig. 1:**
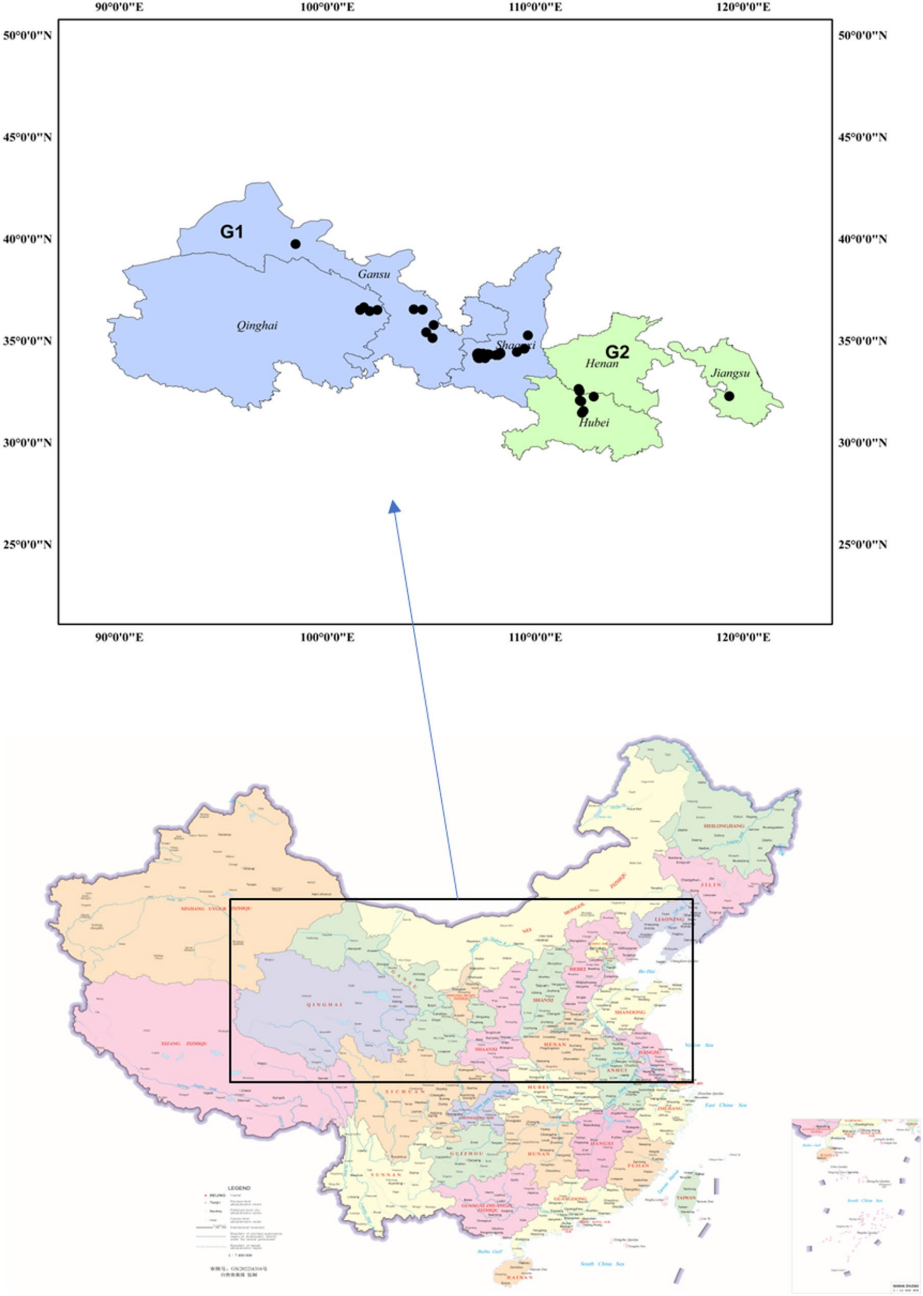
Geographic distribution of *Puccinia striiformis* f. sp. *tritici* collection sites across six Chinese provinces during 2023–2024. The two epidemic regions (G1 and G2) are G1 (Group1-blue)—Northwestern regions (Gansu, Qinghai, and Shaanxi), and G2 (Group2-green)- Central and Eastern regions (Hubei, Henan, and Jiangsu), source of map http://bzdt.ch.mnr.gov.cn/ and ARC GIS 10.8

Stripe rust samples were collected from wheat fields across six provinces representing two major epidemic regions during the 2023–2024 growing season. The provinces included Gansu (GS), Qinghai (QH), and Shaanxi (SX) from the northwestern region (Group1-G1), and Hubei (HB), Henan (HN), and Jiangsu (JS) from the central-eastern region (Group2-G2) (Fig. 1). Sample collection followed established protocols [9], Five to ten leaves with only a single stripe pustule were collected from each field and placed in paper bags. The numbers of isolates from each location of the provinces are given in Supplementary Table S1. At the laboratory, the samples were transferred into new bags and stored at room temperature (20 to 25 °C) for 1 or 2 days to dry completely. The dried samples were kept in a desiccator in a refrigerator (4 °C) for later use.

### Isolate recovery and multiplication

Winter wheat Mingxian 169 (MX169), susceptible to all *Pst* races, was used to recover and multiply urediniospores. Before inoculation, the collected wheat leaves with *Pst* uredinia were washed with water, then placed in Petri dishes with a moist filter paper, and put in a dark dew chamber (10 °C and 100% relative humidity) for 8 to 10 h. Then, individual uredinia were picked and transferred onto MX169 leaves at the two-leaf stage with sterile pins, one uredinium on one leaf. The inoculated wheat seedlings were placed in a plastic box and incubated in a dark dew chamber (10 °C and 100% relative humidity) for 24 h and then transferred to a greenhouse at 16/13 °C (day/night) with fluorescent light for 17 h every day [42]. When the inoculated leaves had chlorotic blotches, only one infected plant was kept for each sample for sporulation by cutting off the other plants in the pot. The inoculated wheat seedlings were isolated with transparent plastic covers to avoid cross-contamination. Urediniospores produced on one inoculated leaf were collected into a glass tube. The collected fresh urediniospores were used to make a spore suspension in Novec 7100 (a specialized engineered fluid used as a suspension medium for urediniospore preparation, Mining and Manufacturing Company, Maplewood, MN, U.S.A.) and used to inoculate 10-day-old MX169 seedlings for urediniospore multiplication. The inoculation and growth of the inoculated plants were the same as described above. The newly produced urediniospores were collected into glass tubes and kept dry at 4 °C in a desiccator. For long-term storage, the glass tubes containing urediniospores were sealed and stored in a − 80 °C freezer.

### Identification of races and determination of their frequencies and distributions

The *Pst* isolates were tested on the 19 Chinese differentials including Trigo Eureka, Fulhard, Lutescens 128, Mentana, Virgilio, Abbondanza, Early Premium, Funo, Danish 1, Jubilejina II, Fengchan 3, Lovrin 13, Kangyin 655, Suwon 11, Zhong 4, Lovrin 10, Hybrid 46, *Triticum spelta* Album, and Guinong 22, representing diverse resistance backgrounds and regional breeding preferences. Each race designation (e.g., CYR34, CYR32, etc.) represents a specific virulence pattern against these 19 Chinese differential hosts. The single-gene system utilized 18 near-isogenic lines each carrying individual *Yr* resistance factors including *Yr1*, *Yr6*, *Yr7*, *Yr8*, *Yr9*, *Yr10*, *Yr17*, *Yr24*, *Yr27*, *Yr32*, *Yr43*, *Yr44*, *YrSP*, *YrTr1*, *YrExp2*, and *Yr76* in standardized susceptible backgrounds (Supplementary Table S3) as previously described [9, 35, 36]. Races identified through the *Yr* single-gene differential set were designated with VR (Virulence Race) nomenclature. In this system, each race is assigned a sequential number (VR1, VR2, and VR3) based on unique virulence profile against the 18 near-isogenic lines. The differentials were planted in pots (7 × 7 × 9 cm), and each pot contained four differentials, one at each corner. MX169 was used as susceptible control. Seedlings at the two-leaf stage were sprayed with 0.1% Tween-20 before inoculation. Fresh urediniospores mixed with talc at a 1:20 ratio was used to inoculate the differentials. Inoculation and growth of the plants after inoculation were the same as described above. After MX169 had full sporulation, the infection types (ITs) were recorded according to the 0 to 9 scale [23, 37]. IT data were converted to avirulence or virulence to describe the virulence–avirulence patterns. An isolate was considered avirulent on a specific differential when it produced an IT of 0 to 6 or virulent when it produced an IT of 7 to 9 [11]. The Virulence Analysis Tool (VAT) [19] was used to analyze the data and calculate the frequencies and distributions of races.

### Determination of virulence frequencies and distributions

Based on the virulence or avirulence profiles of the isolates on the Chinese and *Yr* single-gene differentials, the virulence frequencies and distributions in each province and epidemic region were calculated using the Virulence Analysis Tool (VAT) and Excel (Microsoft, Redmond, WA, U.S.A.). Virulence frequencies were classified as high (> 60%), moderate (20 to 60%), and low (< 20%) [10].

### Clustering analysis

To determine the relationships among the provincial populations, the *Pst* isolates collected from 6 provinces were clustered using the *poppr* package in the R program [16] based on *Nei’s* genetic distance and calculated the multiple diversity indices to capture different aspects of population diversity: Shannon diversity index (overall diversity), Nei’s diversity index (genetic diversity), Kosman index (pathogen virulence diversity), Simpson index (dominance patterns), Shannon evenness index, and Stoddart index [20, 27, 44]. DAPC was selected as it does not assume Hardy–Weinberg equilibrium, making it appropriate for clonal pathogen populations. Discriminant analysis of principal components (DAPC) was implemented in the ADEGENET package in R environment [15] to infer on the clustering pattern based on their virulent profile and assess the grouping in relation to sampling locations. The number of groups was identified based on the Bayesian Information Criterion (BIC), as suggested by Jombart et al. [15]; the assignment of different isolates to these groups was presented in the form of bar chart.

## Results

### Race diversity and distribution patterns using Chinese differential varieties

A total of 54 *Pst* races, including 23 previously reported races and 31 new races, were identified from the 209 isolates using the 19 Chinese differentials. Among these, the most predominant races were CYR34 (19.62%), CYR32 (10.53%), CYR33 (9.09%), and G22-14 (7.66%), with the new 31 races representing 57.4% diversity. these four races were detected in all four epidemic regions; and these newly discovered types representing 20.62% of all pathogen evolution within Chinese populations. These races were identified from the 209 isolates using the 19 Chinese differentials. The differential races CYR34, CYR32, CYR33, G22 breeding derivatives, and international reference races demonstrated similar regional clustering with Hubei and Shaanxi maintaining the highest pathogen diversity (29 and 30 races respectively, total 75 and 72 isolates). CYR34 emerged as the most widespread race across provinces, with peak frequencies in Hubei (14), Jiangsu (10), and Shaanxi (9), while CYR32 and CYR33 showed regional preferences for northern provinces, particularly Shaanxi (9 and 8 isolates respectively) (Table 1). Significant regional differences in race distribution were observed, with CYR34 predominant in central-eastern regions (G2), while CYR32 and CYR33 showed preference for northwest regions (G1). Eight races (CYR28, G22-13, Su11-1, Su11-086, Su11-10, HY-007–1, G22-349, and HY-6) had frequencies of 1.44 to 2.87%, while the remaining 10 known races had frequencies of less than 1%. Among the 31 new races (New1 to New31), 3 (New1 to New3) had frequencies of more than 1%, and 25 (New7 to New31) each were identified from a single isolate. High virulence frequencies (> 60%) were observed for 17 of the 19 Chinese differentials, indicating widespread ineffectiveness of these resistance sources.

**Table 1:** Races of *Puccinia striiformis* f. sp. *tritici* detected using 19 Chinese differential hosts and their virulence binary formulae and octal codes, number of isolates, frequencies, and distributions in various regions

**Races **	**Virulence profile**	**Octal code**	**No. of isolates**	**Frequency (%)**	**Regions (%)**^**a**^
CYR34	1111111111111101101	1777755	41	19.62	G1 (39.02), G2 (60.10)
CYR32	1111111111111101100	1777754	22	10.53	G1 (59.10), G2 (40.10)
CYR33	1111111111111101000	1777750	19	9.09	G1 (63.20), G2 (36.84)
G22-14	1111111111111101001	1777751	16	7.66	G1 (37.50), G2 (62.52)
CYR31	1111111110110101100	1776654	15	7.18	G1 (40.00), G2 (60.00)
CYR28	1111111110100001000	1776410	6	2.87	G1 (50.00), G2 (66.72)
G22-13	0110011111111101101	0637755	7	3.35	G1 (57.14), G2 (28.61)
Su11-1	1100001000000100000	1410040	5	2.39	G1 (50.00), G2 (50.00)
Sul1-086	0111011101100100000	0735440	6	2.87	G1 (60.00), G2 (40.00)
Su11-10	0111011110100100000	0736440	5	2.39	G1 (20.00), G2 (80.00)
HY-007-1	0111111111111101100	0777754	4	1.91	G1 (25.00), G2 (75.00)
G22-349	0011011111001100001	0337141	4	1.91	G1 (50.00), G2 (50.00)
Hy-6	1111111110100100100	1776444	3	1.44	G1 (33.33), G2 (66.71)
Hy-4	1111111111101101100	1777554	2	0.96	G1 (50.00), G2 (50.00)
Lov10-2	1110011110100001000	1636410	2	0.96	G2
Lov13-8	1111111111110001000	1777610	1	0.48	G1
Sul1-389	0011101101111101000	0355750	2	0.96	G2
CYR24	1111011100100000000	1734400	1	0.48	G1
HY-184	0111111111111001100	0777714	1	0.48	G1
Sul1-164	1110111011101010000	0735650	1	0.48	G1
Lov13-2	1111011110110001000	1736610	1	0.48	G2
G22-133	1111111111100100101	1777445	1	0.48	G2
Su11-8	1111011110100100000	1736440	1	0.48	G2
New1	1010100010100101001	1242451	5	2.39	G1 (20.00), G2 (80.00)
New2	0110010010111001100	0622714	4	1.91	G1 (50.00), G2 (50.00)
New3	1010111101010001100	1275214	3	1.44	G1 (33.33), G2 (66.70)
New4	0001010101000101100	0125054	2	0.96	G1
New5	1011001010010001100	1312214	2	0.96	G1 (50.00), G2 (50.00)
New6	1111001000110001000	1710610	2	0.96	G1
New7	1110010000100100001	1620441	1	0.48	G1
New8	0000111011100100000	0073440	1	0.48	G2
New9	1111111111111101001	1777751	1	0.48	G1
New10	1000110101100101000	1065450	1	0.48	G1
New11	0111100101011001001	0745311	1	0.48	G1
New12	1111100111001100101	1747145	1	0.48	G1
New13	0001010000111101100	0120754	1	0.48	G2
New14	1100001001011100101	1411345	1	0.48	G1
New15	1010000111111000001	1207701	1	0.48	G1
New16	0101010001111000001	0521701	1	0.48	G1
New17	1011100010110100000	1342640	1	0.48	G2
New18	1000010001001100000	1021140	1	0.48	G2
New19	1100011010100000000	1432400	1	0.48	G1
New20	0010100110000101001	0246051	1	0.48	G1
New21	1000100010100000100	1042404	1	0.48	G2
New22	0111100000111001000	0740710	1	0.48	G1
New23	1110001001000101000	1611050	1	0.48	G2
New24	0100000000110100101	0400645	1	0.48	G1
New25	1110001001111100101	1611745	1	0.48	G2
New26	1100110000010001000	0146021	1	0.48	G1
New27	0001011101000000000	0135000	1	0.48	G2
New28	0010010101110001100	0225614	1	0.48	G2
New29	1011100101110001000	1345610	1	0.48	G1
New30	1001001110110001101	1116615	1	0.48	G1
New31	1101011010001001000	1532110	1	0.48	G2

*G1* Northwestern regions, including, *GS* Gansu, *QH* Qinghai, *SX* Shaanxi, *G2* Central and Eastern regions, *HB* Hubei, *HN* Henan, *JS* Jiangsu

^a^Races without showing frequencies were identified from only one region, as most of the races were associated with a single isolate

**Fig. 2:**
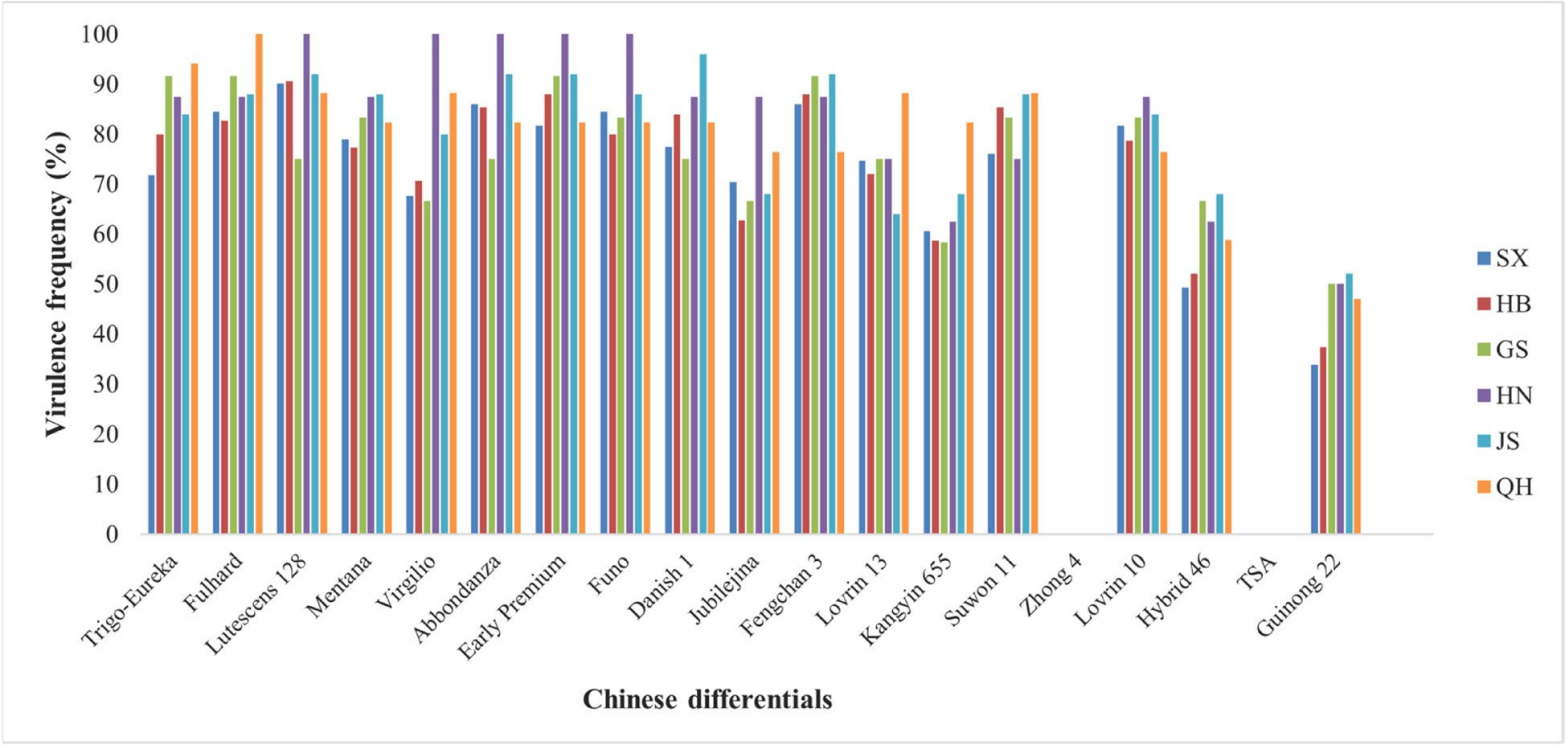
Virulence frequency distributions against 19 Chinese differential wheat varieties across six provinces (Shaanxi = SX, Hubei = HB, Gansu = GS, Henan = HN, Jiangsu = JS, Qinghai = QH)

High virulence frequencies (> 60%) were observed for 17 to 19 Chinese differentials, with Lutescens 128 showing highest susceptibility (Fig. 2; Supplementary Table S4). Only Zhong 4 and *T. spelta Album* showed complete effectiveness (0% virulence frequency) across all tested isolates. Great variations in virulence frequency were found among provinces for most of the differentials, especially Kangyin 655, Fengchan 3, Abbondanza, Lovrin 10, Jubilejina II, Lovrin 13, Hybrid 46, Guinong 22. It is worth pointing out that the frequencies of virulence factors on Virgilio in Shaanxi and Gansu were lower than those in other provinces, the frequencies of virulence JubilejinaII in Gansu, Hubei, Jiangsu were lower than those in other provinces, and the frequencies of virulence Lovrin 13 were lower than those in other provinces.

### Frequencies and distributions of races and virulence factors identified using the Yr single-gene differentials

#### Race frequencies and distributions

From the 209 isolates analyzed using the 18 *Yr* single-gene differentials, a total of 63 races were identified and designated as VR1 to VR63 (Virulence Race1 to Virulence Race63). The VR races, representing long-term pathogen evolution, distributed primarily in Hubei (35 races, 75 isolates) and Shaanxi (34 races, 72 isolates), with moderate presence in Jiangsu (20 races, 25 isolates), Qinghai (13 races, 17 isolates), and lower diversity in Gansu (11 races, 12 isolates) and Henan (7 races, 8 isolates). The traditional VR races VR1, VR2, and VR3 exhibited broad geographic distribution with VR1 being most prevalent in Shaanxi (9) and Hubei (8) (Table 2). VR1 was the most predominant race with a frequency of 10.05% and detected across multiple provinces.

**Table 2:** Race identification results for *Puccinia striiformis* f. sp. *tritici* using 18 *Yr* single-gene differential wheat varieties, including race designations, binary virulence patterns, octal codes, isolate numbers, frequency percentages, and regional

**VR**^**a**^** races**	**Virulence profile**	**Octal code**	**N**^**b**^	**Freq (%)**	**Provinces (%)**^**c**^	**Regions (%)**^**c**^
VR1	1011111000010010011	574111	21	10.05	QH (9.52), SX (42.85), HN (4.76), HB (38.09), JS (4.76)	G1 (52.42), G2 (47.61)
VR2	1011110001110010111	570713	13	6.22	GS (7.69), QH (15.38), SX (38.46), HB (23.07), JS (15.38)	G1 (61.53), G2 (38.45)
VR3	1011110011010010101	571512	12	5.74	GS (8.33), SX (33.33), HB (33.33), JS (25.00)	G1 (41.65), G2 (58.33)
VR4	1011100011101010011	561651	12	5.74	SX (33.33), HN (16.66), HB (41.66), JS (8.33)	G1 (33.33), G2 (66.72)
VR5	0001111010000000101	075002	11	5.26	SX (27.27), HN (9.09), HB (63.63)	G1 (27.33), G2 (72.72)
VR6	0011011010000010011	155011	9	4.31	GS (11.11), SX (22.22), HN (11.11), HB (44.44), JS (11.11)	G1 (33.33), G2 (66.65)
VR7	1000101001010000101	424502	7	3.35	QH (14.28), SX (14.28), HB (42.85), JS (28.57)	G1 (28.56), G2 (71.42)
VR8	1010110001100000111	530603	7	3.35	GS (14.28), SX (42.85), HB (28.57), JS (14.28)	G1 (57.14), G2 (42.85)
VR9	0011000001010100111	140523	7	3.35	SX (57.14), HB (42.85)	G1 (57.14), G2 (42.85)
VR10	1010110000010010101	530112	6	2.87	QH (33.33), SX (33.33), HB (33.33)	G1 (66.66), G2 (33.33)
VR11	1011010010000010011	551011	6	2.87	QH (16.66), SX (33.33), HB (33.33), JS (16.66)	G1 (50.00), G2 (50.00)
VR12	1011111010101110101	575272	4	1.91	SX	G1
VR13	0010100000001000111	120043	4	1.91	HB (75.00), JS (25.00)	G2
VR14	1011110010101010111	571253	4	1.91	QH (33.33), SX (50.00)	G1
VR15	0011100010010010101	161112	4	1.91	SX 50.00), HB (50.00)	G1 (50.00), G2 (50.00)
VR16	1001011000100010101	454212	3	1.44	HB (66.66), JS (33.33)	G2
VR17	1011100010010110011	561131	3	1.44	SX (66.66), JS (33.33)	G1 (66.66), G2 (33.33)
VR18	1011110000011110111	570173	3	1.44	QH (33.33), SX (33.33), HB (33.33)	G1 (66.66), G2 (33.33)
VR19	1011110010101110111	571373	3	1.44	GS (33.33), SX (33.33), HB (33.33)	G1 (66.66), G2 (33.33)
VR20	1011111001100110111	574633	3	1.44	QH (33.33), SX (33.33), HB (33.33)	G1 (66.66), G2 (33.33)
VR21	1011100000001010101	560052	3	1.44	HB	G2
VR22	1011100000000000011	560001	3	1.44	QH (33.33), SX (33.33), HB (33.33)	G1 (66.66), G2 (33.33)
VR23	1010111000001000101	534042	3	1.44	GS (33.33), HB (33.33), JS (33.33)	G1 (33.33), G2 (66.66)
VR24	0011110000000010011	170011	3	1.44	GS (66.66), HB (33.33)	G1 (66.66), G2 (33.33)
VR25	1001010000010010111	450113	3	1.44	QH (33.33), SX (66.66)	G1
VR26	1011110000000010101	570012	3	1.44	SX	G1
VR27	1011011001011110101	554572	3	1.44	HB	G2
VR28	0011111000111010111	174353	2	0.96	JS	G2
VR29	0011100000110011011	160315	2	0.96	SX (50.00), HB (50.00)	G1 (50.00), G2 (50.00)
VR30	1011110000100000111	570203	2	0.96	SX (50.00), HB (50.00)	G1 (50.00), G2 (50.00)
VR23	1010111000001000101	534042	3	1.44	GS (33.33), HB (33.33), JS (33.33)	G1 (33.33), G2 (66.66)
VR24	0011110000000010011	170011	3	1.44	GS (66.66), HB (33.33)	G1 (66.66), G2 (33.33)
VR25	1001010000010010111	450113	3	1.44	QH (33.33), SX (66.66)	G1
VR26	1011110000000010101	570012	3	1.44	SX	G1
VR27	1011011001011110101	554572	3	1.44	HB	G2
VR28	0011111000111010111	174353	2	0.96	JS	G2
VR29	0011100000110011011	160315	2	0.96	SX (50.00), HB (50.00)	G1 (50.00), G2 (50.00)
VR30	1011110000100000111	570203	2	0.96	SX (50.00), HB (50.00)	G1 (50.00), G2 (50.00)
VR31	1010110000000100111	530023	2	0.96	GS (50.00), SX (50.00)	G1
VR32	1011000011100010101	541612	2	0.96	GS (50.00), HN (50.00)	G1 (50.00), G2 (50.00)
VR34	0010011000010010101	114112	2	0.96	GS (50.00), HB (50.00)	G1 (50.00), G2 (50.00)
VR35	1011110001001011101	570456	2	0.96	SX	G1
VR36	1011111011110010101	575712	2	0.96	SX (50.00), HB (50.00)	G1 (50.00), G2 (50.00)
VR37	1011100000001110001	560070	2	0.96	GS (50.00), HN (50.00)	G1 (50.00), G2 (50.00)
VR38	0011011000001000111	154043	1	0.48	QH	G1
VR39	1011111000011010111	574153	1	0.48	QH	G1
VR40	0010010010001000001	111020	1	0.48	SX	G1
VR41	1001000010000000011	441001	1	0.48	HB	G2
VR42	1000110000010100001	414050	1	0.48	HB	G2
VR43	0001110000001000101	070042	1	0.48	HB	G2
VR44	0010100000100000001	120100	1	0.48	HB	G2
VR45	1011010000000000011	550001	1	0.48	SX	G1
VR46	0010010001000100101	110422	1	0.48	JS	G2
VR47	1010000001010000011	500501	1	0.48	JS	G2
VR48	1010100000101000001	520240	1	0.48	SX	G1
VR49	1000000000110010001	400310	1	0.48	HB	G2
VR50	0001010000010000111	050103	1	0.48	SX	G1
VR51	0001001011000000001	045400	1	0.48	HB	G2
VR52	0000001010000100011	005021	1	0.48	SX	G1
VR53	0010011001000000011	114401	1	0.48	JS	G2
VR54	1000000001101000001	400640	1	0.48	HN	G2
VR55	0000000001010110001	000530	1	0.48	SX	G1
VR56	0001010010100000001	051200	1	0.48	SX	G1
VR57	1010000000011000011	500141	1	0.48	HB	G2
VR58	1000000000110000101	400302	1	0.48	SX	G1
VR59	0010100001001000001	120440	1	0.48	HB	G2
VR60	0000010010110100001	011320	1	0.48	QH	G1
VR61	0001000011100000011	041601	1	0.48	JS	G2
VR62	0001010000000010001	050010	1	0.48	JS	G2
VR63	1000010000100100001	410220	1	0.48	JS	G2

^a^*VR* Virulence Race

^b^*N* number of isolates

^c^The frequencies of races are indicated in parentheses. For races detected exclusively in a single province and epidemic region, their frequencies of 100% are not shown, as majority of these races were identified by only one isolate. The provinces in each epidemic region are as follows: *G1* Northwestern regions, including, *GS* Gansu, *QH* Qinghai, *SX* Shaanxi, *G2* Central and Eastern regions, *HB* Hubei, *HN* Henan, *JS* Jiangsu

### Race composition and variation within epidemic Regions

The race composition varied considerably between the two major epidemic regions studied based on geographic locations (Fig. 1). Regions of G1 (Northwestern regions: Gansu, Qinghai, Shaanxi) contained 48.32% of the total isolates, with VR1 as the predominant race (10.89% frequency). The top races in G1 included VR1 (10.89%), followed by VR2 (7.92%), VR3 (4.95%), and several other races including VR4, VR8, VR9, VR10, VR12, and VR14 each contributing 3.96% to the regional composition. The northwestern region showed a diverse race structure with multiple races sharing similar frequencies, indicating a complex pathogen population adapted to the over summering conditions typical of this region. In contrast, regions of G2 (Central and eastern regions: Hubei, Henan, Jiangsu) comprised 51.67% of isolates, where VR1 remained predominant but at a slightly lower regional frequency of 9.25%. The G2 regions displayed a different race hierarchy with both VR4 and VR5 at 7.40%, followed by VR3 at 6.48%, demonstrating distinct regional race composition patterns (Table 3). G1 regions showed high virulence frequencies and have high range of races. This was followed by VR2 (6.22%) found in GS (7.69%), QH (15.38%), SX (38.46%), HB (23.07%), and JS (15.38%) provinces, VR3 (5.74%) present in GS (8.33%), SX (33.33%), HB (33.33%), and JS (25.00%), and VR4 (5.74%) detected in SX (33.33%), HN (16.66%), HB (41.66%), and JS (8.33%). VR5 completed the top five races with a frequency of 5.26%, being found in SX (27.27%), HN (9.09%), and HB (63.63%). The remaining 58 races exhibited frequencies below 5%, VR6 and VR7 were the most widely distributed races, as VR6 was detected in five provinces GS (11.11), SX (22.22), HN 11.11), HB (44.44), JS (11.11), and VR7 was detected also QH (14.28), SX (14.28), HB (42.85), JS (28.57) although they had a relatively low frequency (3.35%) compared with VR1 to VR5. The frequencies of VR8 to VR27 ranged from 1.44 to 3.35% and they were detected in 2 or more of the 4 provinces except Henan. The frequencies of VR28 to VR63 were less than 1%, of which 26 races each were detected from only one isolate, 10 races were detected from 2 isolates, and 16 races were detected from 3 (1.44%) to 4 (1.91%) isolates. Of the 63 VR races, 30 were detected in 2 to 6 provinces, while 33 were detected in only 1 province.

**Table 3:** Provincial summary statistics and distributions for VR races identified using 18 *Yr* single-gene differentials across two epidemic regions

Epidemic regions	Number of isolates	Freq (%)	VR (Virulence race) and frequency (%)^a^
G1	101	48.32	**1 (10.89),** 2 (7.92), 3 (4.95), 4, 8, 9, 10, 12, 14 (3.96), 5, 6, 11, 25, 26 (2.97), 7, 15, 16, 17, 18, 19, 20, 21, 22, 31, 35 (1.98), 23, 24, 29, 30, 32, 34, 36,37, 38, 39, 40, 45, 48, 50, 52, 55, 56, 58, 60
G2	108	51.67	**1 (9.25),** 4, 5 (7.40), 3 (6.48), 6 (5.55), 2, 7 (4.62), 13 (3.70), 8, 9, 11, 16, 21, 27 (2.77), 10, 15, 23, 28, 33 (1.85), 17, 18, 19, 20, 22, 24, 29, 30, 32, 34, 36, 37, 41, 42, 43, 44, 46, 47, 49, 51, 53, 54, 57, 59, 61, 62, 63

*G1* Northwestern regions, including, *GS* Gansu, *QH* Qinghai, *SX* Shaanxi, *G2* Central and Eastern regions, *HB* Hubei, *HN* Henan, *JS* Jiangsu

^a^The most predominant race is highlighted in bold for epidemic regions. Races listed without frequencies values each were detected from only one isolate

### Race composition and variation within Provinces

Provincial variation in race composition was particularly striking across the six provinces examined, reflecting local adaptation and environmental pressures on the pathogen population. Shaanxi (SX) exhibited the most complex race structure with races distributed across both epidemic regions (Fig. 5), where VR1 maintained its dominance at 11.8% frequency, followed by VR2 (6.10%), and multiple races including VR3, VR9, and VR12 each at 5.6%. The high diversity in SX suggests this province serves as a crucial epidemiological bridge between the two regions. Hubei (HB) showed considerable diversity with 35 different races identified, with VR1 as the most frequent at 10.7%, followed by VR5 (9.4%) and VR4 (6.7%), indicating strong pathogen pressure and diverse selection forces in this central province. Jiangsu (JS) revealed unique patterns among the 20 races detected, with VR3 being the predominant race at 12%, followed by VR2, VR7, and VR28 each at 8%, Henan (HN) showed more limited diversity with only 7 races but had VR4 as the dominant race at 25%, indicating either strong selection pressure for this particular race, Gansu (GS) presented 11 races with VR24 being most prevalent at 16.66%, while Qinghai (QH) displayed 13 races with VR1, VR2, VR10, and VR14 each sharing dominance at 11.76% (Table 4). Of the six provinces, Hubei had the highest number of races (35), followed by Shaanxi (34), Jiangsu (20), and Qinghai (13), Gansu (11). However, HB had the highest race/isolate ratio (1:2.14), followed by SX (1:2.11), JS (1: 1.25), QH (1:1.30), and (1:1.09).

**Table 4:** Number of *Puccinia striiformis* f. sp. *tritici* isolates, number of races, and VR race frequency patterns across various sites using 18 *Yr* single-gene differentials

**Province**^a^	**No. of isolates**	**No. of races**	**VR races and frequency (%)**^**b**^
SX	72	34	**1 (11.8)**, 2 (6.10), 3, 4, 9, 12 (5.6), 5, 8, 26 (4.2), 6, 10, 11, 14, 15, 17, 25, 35 (2.8), 7, 18, 19, 20, 22, 29, 30, 31, 36, 40, 45, 48, 50, 52, 55, 56, 58 (1.4)
HB	75	35	**1 (10.7),** 5 (9.4), 4 (6.7), 3, 6 (5.33), 2, 7, 9, 13, 21, 27 (4), 8, 10, 11, 15, 16 (2.7), 18, 19, 20, 22, 23, 24, 29, 30, 33, 34, 36, 41, 42, 43, 44, 49, 51, 57, 59 (1.33)
GS	12	11	**24 (16.66)** 2, 3, 6, 8, 19, 23, 31, 32, 34, 37 (8.33)
HN	8	7	**4 (25)**, 1, 5, 6, 32, 37, 54 (12.5)
JS	25	20	**3 (12)**, 2, 7, 28 (8), 1, 4, 6, 8, 11, 13, 16, 17, 23, 33, 46, 47, 53, 61, 62, 63 (4)
QH	17	13	**1, 2, 10, 14 (11.76)**, 7, 11, 18, 20, 22, 25, 38, 39, 60 (5.88)

^a^The provinces in each epidemic region are as follow: *G1* Northwestern regions, including, *GS* Gansu, *QH* Qinghai, *SX* Shaanxi, *G2* Central and Eastern regions, *HB* Hubei, *HN* Henan, *JS* Jiangsu

^b^The most predominant race in each province is highlighted in bold. Races listed without frequencies values each were detected from only one isolate

### Virulence frequencies and distributions

**Fig. 3:**
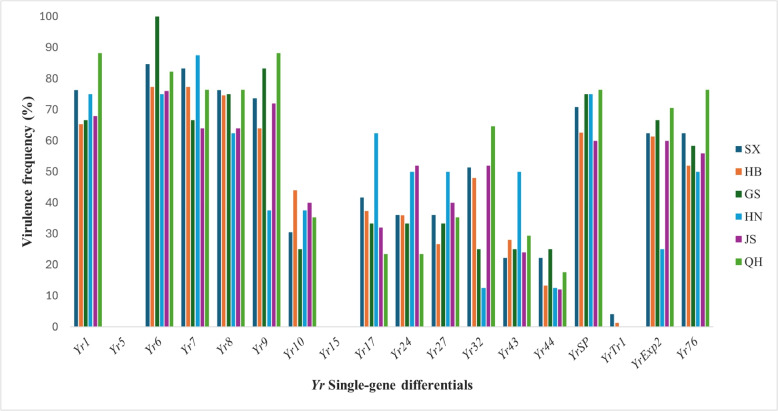
Compatibility frequencies against individual *Yr* single-gene across six provinces, (Shaanxi = SX, Hubei = HB, Gansu = GS, Henan = HN, Jiangsu = JS, Qinghai = QH). showing percentage of isolates capable of overcoming each resistance gene within provincial

The virulence frequency analysis revealed significant spatial variation in the effectiveness of resistance genes across different regions and provinces, indicating complex pathogen-host interactions and varying selection pressures on different *Yr* genes. Based on the race composition and distribution patterns observed in the study, several *Yr* genes would likely demonstrate nationwide virulence frequencies to *Yr1, Yr6, Yr7, Yr9*, *YrSP* and *YrExp2* > 60% across both epidemic regions G1 and G2, indicating that these resistance genes have lost their effectiveness against the majority of the *Pst* population (Fig. 3; Supplementary Table S3). The frequencies of virulence factors to *Yr76, Yr8, Yr32, Yr10, Yr17,* and *Yr27* were moderate (36.84% to 58.00%), and they were detected in all endemic regions and provinces, the low frequency were detected to *Yr43*, *Yr24*, and *Yr44*, (19.61 to 26.31%), except for *YrTr1*, which was not detected in GS, HN, JS, QH provinces. The frequencies of virulence factors to *YrTr1* (1.33 to 4.14%) were low, and they were detected only in SX, HB provinces. All isolates were avirulent to *Yr5* and *Yr15*, indicating that these resistant genes were effective against the *Pst* populations in China.

The frequencies of virulence factors varied greatly among the epidemic regions (Fig. 3; Supplementary Table S3). In G1, high frequencies were found for virulence factors to *Yr1* (88.24%), *Yr6* (84.71%), *Yr7* (83.33%), *Yr9* (88.24%), *Yr8* (76.47%), *YrSP* (76.47%), *Yr76* (76.47%), *YrExp2* (70.59), and *Yr32* (64.71%); moderate frequencies for virulence factors to *Yr17* (41.70%), *Yr24* (36.1%), *Yr27* (36.1%), and *Yr10* (35.29%); and low frequencies for virulence factors to *Yr44* (17.65%), *Yr43* (29.41%). In G2, the frequencies of virulence factors to *Yr7* (87.5%), *Yr6* (77.3%), *Yr1* (75.00%), *YrSP* (75.00%), *Yr8* (74.7%), *Yr9* (72.00%), *Yr17* (62.5%), and *YrExp2* (61.3%) were high; the frequencies of virulence factors to *Yr10* (44.00%), *Yr76* (56.00%), *Yr24* (52.00%), *Yr27* (50.00%), *Yr32* (52.00%) and *Yr43* (50.00%) were moderate; and the frequencies of virulence factors to *Yr44* (13.3%) and *YrTr1* (1.31%) were low. No isolates were virulent to *Yr5* and *Yr15*. The differences in virulence frequency among the 6 provinces are illustrated in Fig. 6. High variations in frequency among these provinces were observed for virulence factors to* Yr17*, *Yr24*, *Yr27*, *Yr43* and *Yr44*. The frequencies of *Yr9* and *YrExp2* in Henan were lower from those of other provinces. For *Yr32* the virulence frequencies of other provinces were lower than 51.38% except for Qinghai (64.70%). For *Yr6*, the virulence frequency in Gansu was 100%, which was different from that of any other province, *YrSP* was not different from Gansu and Henan. The frequencies of virulence factors to *Yr10* in Gansu, Qinghai, Shaanxi, Jiangsu, Henan were less than 44%. For *Yr*7 and *Yr8* was not different from Jiangsu, the virulence frequency in Qinghai *Yr1* and *Yr76* was different from that of any other province.

### Cluster analysis based on the virulence data

**Fig. 4:**
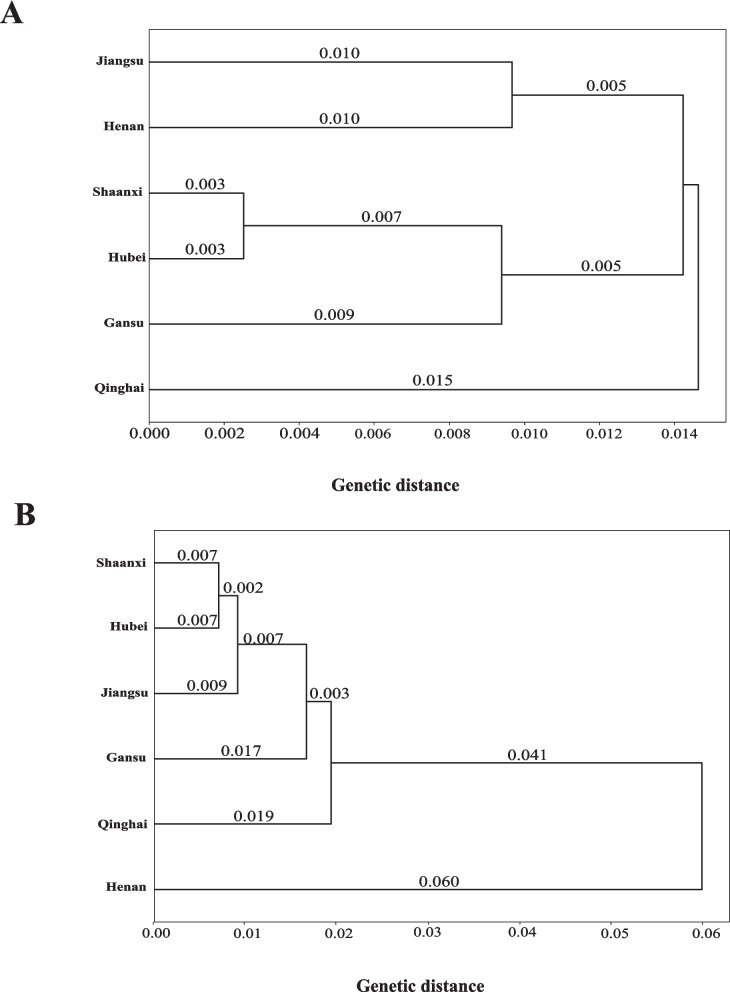
UPGMA phylogenetic trees showing virulence relationships among six provincial populations of *Puccinia striiformis* f. sp. *tritici*. Nei’s genetic distance values on the nodes (**A**), based on 19 Chinese differential. **B**, based on 18 *Yr* single-gene differential

The six provincial *Pst* populations were clustered separately based on the virulence data from the two sets of differentials. The UPGMA dendrogram based on *Nei's* genetic distances using phenotypic responses to 19 Chinese differential hosts revealed distinct patterns of virulence divergence among pathogen populations from six Chinese provinces (Fig. 4A). The 6 populations were clustered into two major groups based on the Chinese differentials. Cluster I comprises Hubei (HB) and Shaanxi (SX) populations, indicating high similarity in virulence phenotypes between these two provinces. Cluster II further subdivides into two sub-clusters: Cluster IIa contains Jiangsu (JS) and Henan (HN) populations, while Cluster IIb groups Gansu (GS) and Qinghai (QH) populations together. The branch lengths in the dendrogram reflect the degree of virulence divergence, with shorter branches indicating greater phenotypic similarity and longer branches suggesting more distinct virulence spectra. Based on virulence profiles on the 18 *Yr* single-gene differentials, the 6 provincial populations were clustered into three groups. and longer branches suggesting more distinct virulence spectra. Based on virulence profiles on the 18 *Yr* single-gene differentials, the 6 provincial populations were clustered into three groups. Cluster A groups Hubei (HB) with Jiangsu (JS), showing moderate virulence similarity against *Yr* genes. Cluster B contains only Shaanxi (SX), appearing as an outlier with distinct virulence characteristics when tested against single resistance genes (Fig. 4B). Cluster C encompasses Henan (HN), Gansu (GS), and Qinghai (QH), with GS and QH maintaining their close relationship from the previous analysis, while HN shows moderate similarity to this western province pair. The reorganization of provincial relationships in this dendrogram indicates that when virulence is assessed against specific *Yr* resistance genes, the clustering pattern differs significantly from the Chinese differential host analysis. Notably, the HB-SX pairing observed in Fig. 4A is disrupted, with SX becoming isolated, while HB forms a new association with JS. The GS-QH pair remains the most similar across both analyses (Table 5).

**Table 5:** Virulence and pathotype diversity of *Puccinia striiformis* f. sp. *tritici* isolates in different geographic population, values show as Chinese differentials/*Yr* single-gene differentials

Parameters^a^						
**Geographic Provinces**	***SH***	***Hs***	***K***	***Si***	***Sh***	***St***
Shaanxi	4.24/5.09	0.308/0.321	0.308/0.321	0.934/0.953	0.891/0.933	15.07/21.073
Hubei	4.37/5.13	0.301/0.337	0.301/0.337	0.922/0.953	0.873/0.929	12.755/21.388
Gansu	2.92/3.46	0.292/0.299	0.292/0.299	0.833/0.903	0.921/0.988	6/10.286
Henan	2.75/2.81	0.196/0.317	0.196/0.317	0.844/0.844	0.98/0.98	6.4/6.4
Jiangsu	3.13/4.32	0.244/0.345	0.244/0.345	0.8/0.941	0.827/0.975	5/16.892
Qinghai	2.96/3.7	0.25/0.282	0.25/0.282	0.872/0.913	0.942/0.977	7.811/11.56

^a^Shannon diversity index = *SH*, Nei’s diversity index = *Hs*, Kosman index = *K*, Simpson index = Si, Shannon evenness index = *Sh*, and Stoddart index = *St*. Values represent diversity indices calculated Chinese differential hosts (left) and *Yr* single-gene differentials (right)

**Fig. 5:**
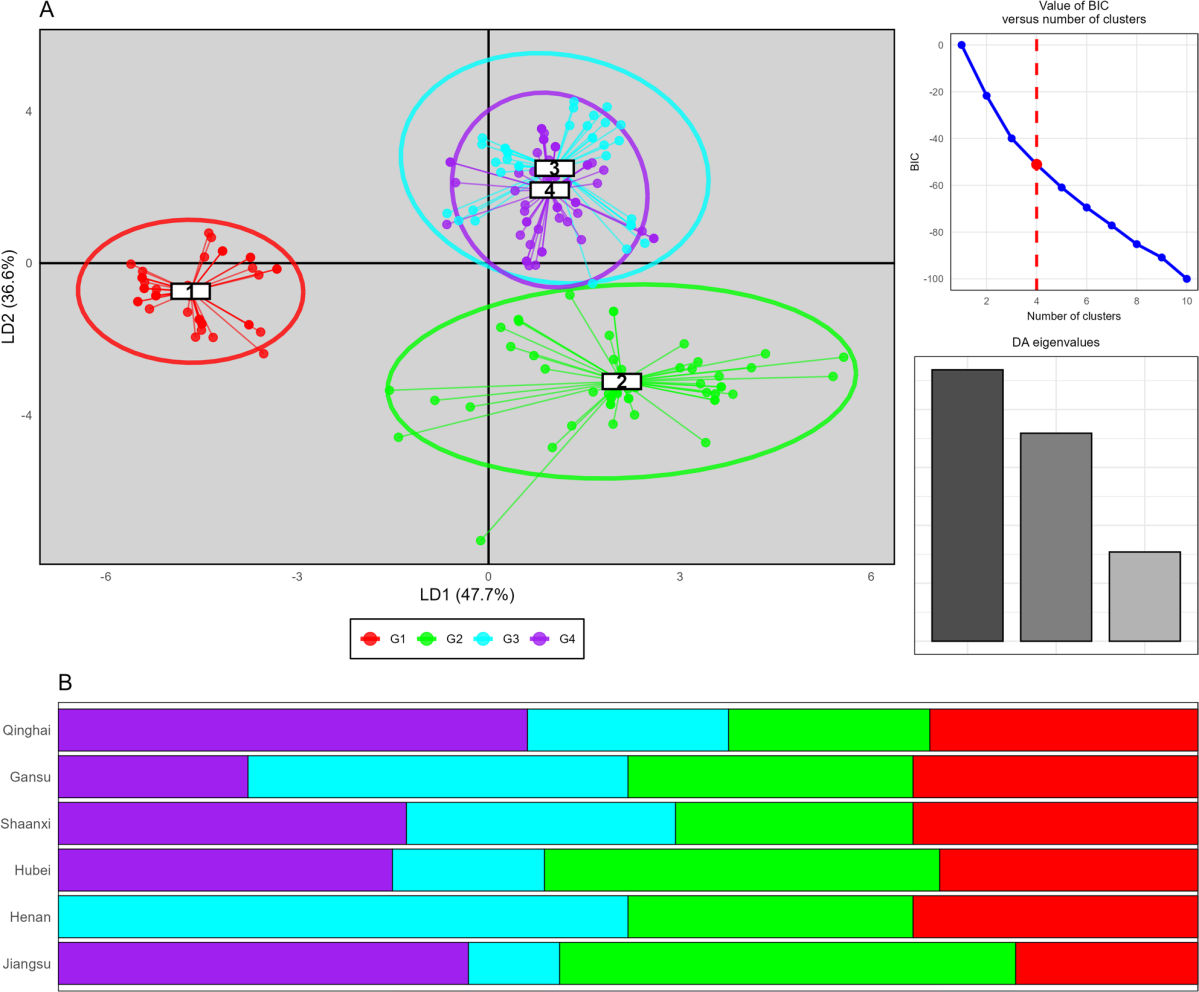
Discriminant analysis of principal components (DAPC) applied to 209 *Puccinia striiformis* f.sp. *tritici* isolates collected from 6 Chinese provinces, revealing four distinct clusters based on virulence patterns against Chinese wheat cultivars and *Yr* resistance genes. **A** DAPC scatter plot displaying the differentiation of isolates into four groups, with optimal cluster number validated by Bayesian Information Criterion (BIC) analysis (upper right panel). **B** Distribution of isolates across 6 geographic locations among the four identified genetic clusters

The non-parametric discriminant analyses of principal components (DAPC) on virulence profile identified four groups (K = 4), as confirmed by the Bayesian information criteria (BIC) curve. However, the grouping was explained by the pathotypes rather than the geographic locations, suggesting frequent migration of the races from these groups across these geographical regions (Fig. 5A). The spatial distribution showed that pathotypes clustering was independent of geographic locations, with isolates from different groups present across multiple provinces, and all four clusters (G1-G4) were present across multiple provinces, with varying proportions (Fig. 5B), supporting frequent pathotypes exchange that challenges traditional assumptions regional pathogen isolation.

### Race differentiation using differential host lines

A large number of isolates of the same races identified using the 19 Chinese differentials were differentiated into different races using the 18 *Yr* single-gene differentials. Analysis of the three most predominant races identified using Chinese differentials revealed that 41 isolates of race.

**Fig. 6:**
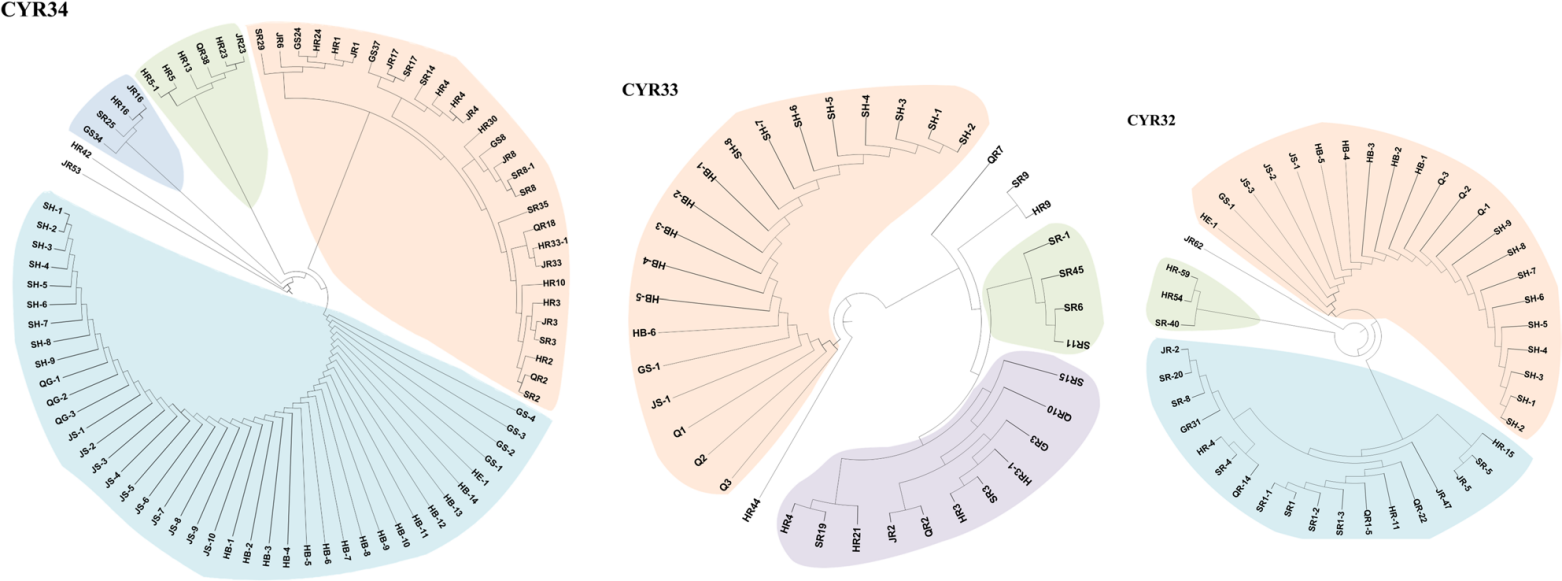
Hierarchical clustering analysis demonstrating virulence subdivisions within major races CYR34, CYR33 and CYR32 that were identified with the Chinese set of differentials further differentiated using the Yr single-gene differentials

CYR34 were differentiated into 25 VR races, the 19 isolates of race CYR33 as 14 VR races, and the 22 isolates of race CYR32 as 16 VR races using the single-gene differentials (Fig. 6). Comparative analysis of Shannon diversity indices revealed systematic differences between the two differential systems across all six provinces. The 18 *Yr* single-gene differential system consistently showed higher diversity values compared to the 19 Chinese differential hosts, with diversity indices ranging from 2.81 to 5.09 for the *Yr*-gene system versus 2.75 to 4.37 for the Chinese differential system. While both systems identified Shaanxi and Hubei as harboring the most diverse pathogen populations and Henan as having the lowest diversity, the *Yr*-gene differentials demonstrated enhanced discriminatory power, detecting an average of 0.25–0.37 additional diversity units per province.

## Discussion

Stripe rust represents one of the most economically significant disease of wheat globally, with China serving as the world's largest epidemic area [8, 33]. Continuous *Pst* evolution necessitates ongoing surveillance for effective resistance deployment strategies [12, 28]. This study's identified 54 races using 19 Chinese differentials and 63 VR races using 18 *Yr* single-gene differentials from 209 isolates across six provinces. The predominance of races CYR34 (19.62%), CYR32 (10.53%), and CYR33 (9.09%) aligns with recent epidemic patterns [45] and continued dominance of the Guinong 22 race group responsible for severe epidemics [26, 42]. The detection of 31 new races (57.4% of the total) indicates rapid pathogen evolution and the accelerating pace of *Pst* evolution in China, consistent with previous observations Chen et al. [4]. Virulence frequency analysis revealed alarming ineffectiveness among the Chinese differential hosts, with 17 of 19 differentials showing high virulence frequencies (> 60%), indicating widespread breakdown of historically important resistance sources.

Most Chinese differentials showed severe compromise (> 80% virulence frequency), with Lutescens 128 most affected (89.95%), indicating loss of discriminatory power for reliable race identification and breeding programs. Additional differentials including Lovrin 13 (73.86%), Virgilio (73.20%) JubilejinaII (68.42%) and Kangyin 655 (62.67%) demonstrated substantial effectiveness loss, while even Hybrid 46 (55.02%), and Guinong 22 (40.19%) showed concerning compromising levels. The complete effectiveness of Zhong 4 and *Triticum spelta* Album against all isolates provides encouraging evidence for durable resistance sources. *Triticum spelta* Album carries *Yr5*, which maintains effectiveness in China despite virulence reports from India, Turkey, Australia, Tajikistan [41], Syria [18], and limited reports from Shaanxi and Qinghai provinces [43]. The unknown resistance gene(s) in Zhong 4 warrant further characterization given their broad-spectrum effectiveness. Regional patterns revealed distinct pathogen structures between the northwestern (G1) and central-eastern (G2) epidemic regions, reflecting the complex epidemiological system described by Chen et al. [4], with region-specific selection pressures evident in race distribution.

The virulence frequency analysis against individual *Yr* genes provides critical information for resistance deployment approaches. High virulence frequencies (> 60%) against *Yr1*, *Yr6*, *Yr7*, *Yr9*, *YrSP*, and *YrExp2* indicate these genes have lost effectiveness and should not be relied upon as sole resistance sources, with national survey findings [45]. Moderate virulence frequencies (36.84–58.00%) for *Yr76*, *Yr8*, *Yr32*, *Yr10*, *Yr17*, and *Yr27* suggest strategic integration potential within gene combination.

Low virulence frequencies against *Yr43* (19.61%), *Yr24* (26.31%), and *Yr44* (22.12%) indicate substantial effectiveness for contemporary breeding programs. Complete avirulence against *Yr5* and *Yr15* provides strong evidence for their continued effectiveness in China. *Yr5* maintains durability despite limited provincial virulence reports [1, 43], while *Yr15* shows greater stability with virulence reported only from Afghanistan [3, 29, 34]. These genes should be prioritized for pyramid development to enhance durability and prevent rapid virulence evolution [7, 35]. Regional variation in virulence frequencies, such as higher *Yr32* frequency in Qinghai (64.70%) versus other provinces (< 51.38%), and the complete virulence to *Yr6* in Gansu suggest regional pathogen adaptation that could inform targeted resistance deployment. Such regional differences emphasize the need for region-specific resistance strategies.

The differentiation of 41 CYR34 isolates into 25 VRs, 22 CYR32 isolates into 16 VRs, and 19 CYR33 isolates into 14 VR races strongly supports the recommendations of Chen et al. [10] for adopting standardized *Yr* single-gene systems for international race monitoring. Hubei's considerable diversity with 35 different virulence races (VRs) indicates strong pathogen pressure, supporting its importance as a key epidemic region [14, 42]. Provincial variation in race composition was striking across the six provinces, with Shaanxi and Hubei exhibiting the most complex race structure and highest Shannon diversity index. High diversity in these provinces suggests they serves as a crucial epidemiological bridge between regions, consistent with their geographic position in China's stripe rust epidemiology [21, 37]. These regional differences reflect the complex epidemiological interactions between autumn, over-summering regions, spring multiplication areas, and epidemic zones that create diverse selective environments for pathogen evolution. Clustering analysis revealed different provincial relationship when assessed against Yr single-gene differentials versus Chinese differential hosts, with Shaanxi emerging as a distinct cluster suggesting its role as an epidemiological bridge between regions due to geographic position and pathogen dispersal patterns. DAPC analysis based on virulence profile identified four distinct groups (G1-G4), with grouping explained by specific virulence combinations (6AB). As the world’s largest wheat stripe rust epidemic area, the virulence structure of *Pst* is complex, and the population genetic diversity is high in China. However, since CYR32 was named in 1994 [38], only two races, CYR33 and CYR34, were named [5, 24].

These findings contribute significantly to understanding of *Pst* population dynamics in China and provide essential baseline data for evidence-based resistance breeding programs. The demonstration of superior discriminatory power of *Yr* single-gene differentials validates their utility for comprehensive pathogen monitoring and supports their broader adoption for sustainable stripe rust management. As the pathogen continues to evolve and adapt to deployed resistance genes, the integration of comprehensive race monitoring with strategic resistance deployment will be crucial for maintaining effective control of this economically important disease. The results support the implementation of enhanced surveillance systems combining both differential sets for sustainable stripe rust management in China and provide a scientific foundation for international cooperation in *Pst* monitoring and resistance gene deployment strategies. Several limitations should be acknowledged. First, continued monitoring will be essential given the pathogen’s rapid evolution. Second, sampling density varied among provinces, potentially affecting regional comparisons. Finally, while this study utilized both differential systems to maximize resolution, some resistance genes in the Chinese differentials set remain genetically uncharacterized, which may limit complete virulence profiling for uncertain isolates.

## Conclusion

From 209 Pst isolates collected across six provinces (2023–2024), we identified 54 races with the Chinese differentials and 63 with the 18 *Yr* single-gene set, confirming the latter’s superior discriminatory power. Predominant races (CYR34, CYR32, CYR33) and 31 new races indicate rapid pathogen evolution, with high virulence to many traditional hosts and to *Yr1, Yr6, Yr7, Yr9, YrSP,* and *YrExp2*, while *Yr5* and *Yr15* remained fully effective. Clear regional contrasts between northwestern (G1) and central-eastern (G2) zones, and high diversity in Shaanxi/Hubei, underscore the need for region-tailored resistance deployment. We recommend routine surveillance using *Yr* single-gene differentials and pyramiding durable genes (prioritizing *Yr5*/*Yr15* with complementary loci) to sustain stripe rust management in China.

## Supplementary Information

Supplementary Material 1.

## Data Availability

All data generated or analyzed during this study are included in this article.

## Abbreviations

BIC: Bayesian Information Criterion
DAPC: Discriminant Analysis of Principal Components
UPGMA: Unweighted Pair Group Method with Arithmetic Mean
VAT: Virulence Analysis Tool
*IT*: Infection Type
G1: Group 1 (Northwestern epidemic region: Gansu, Qinghai, Shaanxi)
G2: Group 2 (Central-Eastern epidemic region: Hubei, Henan, Jiangsu)
CYR: Chinese Yellow Rust (race designation)
VR: Virulence Race (designation system using *Yr* single-gene differentials)
