# Supplementary material for: Virulence and pathotype variability for Puccinia striiformis f. sp. tritici across different geographical regions and epidemic zones of China
Source: BMC Plant Biol. 2026 Feb 5;26:439. doi: 10.1186/s12870-026-08249-8 (PMC12964632; doi:10.1186/s12870-026-08249-8)
Supplement: Supplementary file 1 — Supplementary Material 1. [file 12870_2026_8249_MOESM1_ESM.zip › Supplement Table 1.docx]

| Supplement Table 1. Information of wheat stripe rust samples collected by provinces | | | | | | |
| --- | --- | --- | --- | --- | --- | --- |
| **Year** | **Province** | **City** | **County (District)** | **Latitude and** | **Sample size** | **Total** |
|  |  |  |  | **Longitude** |  |  |
| 2023-2024 |  |  |  |  |  | 209 |
|  | Shaanxi |  |  |  |  | 72 |
|  |  | Baoji | Chencang | 107.454209,34.243811 | 2 |  |
|  |  |  | Chencang | 107.4866, 34.346099 | 3 |  |
|  |  |  | Chencang | 107.38421, 34.172311 | 4 |  |
|  |  |  | Chengcang | 107.345809, 34.26921 | 8 |  |
|  |  |  | Weibin | 107.256909,34.304511 | 9 |  |
|  |  |  | Weibin | 107.28941,34.185309 | 2 |  |
|  |  |  | Taibai | 107.579909, 34.121311 | 7 |  |
|  |  |  | Mei | 107.78362, 34.297349 | 2 |  |
|  |  |  | Mei | 107.71437, 34.314249 | 4 |  |
|  |  |  | Qishan | 107.598863, 34.259033 | 3 |  |
|  |  |  | Wugong | 108.22901, 34.280311 | 2 |  |
|  |  | Xianyang | Wugong | 108.21231, 34.27561 | 3 |  |
|  |  |  | Qian | 108.315521, 34.34291 | 2 |  |
|  |  |  | Qian | 108.308923, 34.385291 | 5 |  |
|  |  |  | Yangling | 108.094499, 34.274012 | 2 |  |
|  |  |  | Baqiao | 109.10871, 34.42521 | 4 |  |
|  |  | Xian | Linwei | 109.47011,34.58451 | 6 |  |
|  |  | Weinan | Baishui | 109.639864,35.240059 | 4 |  |
|  | Hubei | Xiangyang | Yicheng | 112.31,31.531 | 16 | 75 |
|  |  |  | Yicheng | 112.51674,31.747269 | 15 |  |
|  |  |  | Fancheng | 112.136,32.0449 | 9 |  |
|  |  |  | Xiangcheng | 112.201,32 | 6 |  |
|  |  |  | Xiangcheng | 112.19461,32.003682 | 8 |  |
|  |  |  | Zaoyang | 112.803609, 32.234671 | 10 |  |
|  |  | Zhongxian | Jingmen | 112.238463,31.436246 | 11 |  |
|  | Gansu | Jiuquan | Suzhou | 98.467395,39.72518 | 2 | 12 |
|  |  | Dingxi | Anding | 104.749000, 35.393889 | 2 |  |
|  |  |  | Tongwei | 105.053907, 35.110179 | 2 |  |
|  |  | Baiyin | Baiyin | 104.15106,36.519512 | 1 |  |
|  |  |  | Huining | 105.107315, 35.748959 | 2 |  |
|  |  |  | Jingyuan | 104.575162, 36.501713 | 3 |  |
|  | Henan | Dengzhou | Nanyang | 112.0771,32.61 | 5 | 8 |
|  |  |  | Nanyang | 112.12374,32.503411 | 3 |  |
|  | Jiangsu | Yangzhou | Yizheng | 119.31791, 32.25351 | 11 | 25 |
|  |  |  | Yizheng | 119.32131, 32.25571 | 14 |  |
|  | Qinghai | Haidong | Ledu | 102.39990, 36.48839 | 3 |  |
|  |  |  | Ping'an | 102.03205, 36.43251 | 2 |  |
|  |  | Xining | Huangzhong | 101.57334, 36.49347 | 3 | 17 |
|  |  |  | Chengxi | 101.75202, 36.62133 | 6 |  |
|  |  |  | Huangyuan | 101.25400,36.68084 | 3 |  |
