# Supplementary material for: Virulence and pathotype variability for Puccinia striiformis f. sp. tritici across different geographical regions and epidemic zones of China
Source: BMC Plant Biol. 2026 Feb 5;26:439. doi: 10.1186/s12870-026-08249-8 (PMC12964632; doi:10.1186/s12870-026-08249-8)
Supplement: Supplementary file 1 — Supplementary Material 1. [file 12870_2026_8249_MOESM1_ESM.zip › Supplement Table 4.docx]

|  |  |  | **Frequency (%)** | | |  | | |
| --- | --- | --- | --- | --- | --- | --- | --- | --- |
|  | **Country** |  | **G1** | | | **G2** | | |
| **Virulence** | **N** | **Freq. (%)** | **SX** | **GS** | **QH** | **HN** | **HB** | **JS** |
| Trigo-Eureka | 167 | 79.90 | 71.8 | 91.7 | 94.1 | 87.5 | 80.0 | 84 |
| Fulhard | 180 | 86.12 | 84.5 | 91.7 | 100.0 | 87.5 | 82.7 | 88 |
| Lutescens 128 | 188 | 89.95 | 90.1 | 75.0 | 88.2 | 100 | 90.7 | 92 |
| Mentana | 168 | 80.38 | 78.9 | 83.3 | 82.4 | 87.5 | 77.3 | 88 |
| Virgilio | 153 | 73.20 | 67.6 | 66.7 | 88.2 | 100 | 70.7 | 80 |
| Abbondanza | 180 | 86.12 | 85.9 | 75.0 | 82.4 | 100 | 85.3 | 92 |
| Early Premium | 181 | 86.60 | 81.7 | 91.7 | 82.4 | 100 | 88.0 | 92 |
| Funo | 175 | 83.73 | 84.5 | 83.3 | 82.4 | 100 | 80.0 | 88 |
| Danish 1 | 173 | 82.77 | 77.5 | 75.0 | 82.4 | 87.5 | 84.0 | 96 |
| JubilejinaII | 143 | 68.42 | 70.4 | 66.7 | 76.5 | 87.5 | 62.7 | 68 |
| Fengchan 3 | 182 | 87.08 | 85.9 | 91.7 | 76.5 | 87.5 | 88.0 | 92 |
| Lovrin 13 | 154 | 73.68 | 74.6 | 75.0 | 88.2 | 75 | 72.0 | 64 |
| Kangyin 655 | 131 | 62.67 | 60.6 | 58.3 | 82.4 | 62.5 | 58.7 | 68 |
| Suwon 11 | 172 | 82.29 | 76.1 | 83.3 | 88.2 | 75 | 85.3 | 88 |
| Zhong 4 | 0 | 0 | 0 | 0 | 0 | 0 | 0 | 0 |
| Lovrin 10 | 169 | 80.86 | 81.7 | 83.3 | 76.5 | 87.5 | 78.7 | 84 |
| Hybrid 46 | 115 | 55.02 | 49.3 | 66.7 | 58.8 | 62.5 | 52.0 | 68 |
| *Triticum spelta var. Album* | 0 | 0 | 0 | 0 | 0 | 0 | 0 | 0 |
| Guinong 22 | 84 | 40.19 | 33.8 | 50.0 | 47.1 | 50 | 37.3 | 52 |

**Supplementary Table 4.** Virulence frequencies of *Puccinia striiformis* f. sp. *tritici* to 19 Chinese differential hosts countrywide and in various provinces of China
