# Supplementary material for: Virulence and pathotype variability for Puccinia striiformis f. sp. tritici across different geographical regions and epidemic zones of China
Source: BMC Plant Biol. 2026 Feb 5;26:439. doi: 10.1186/s12870-026-08249-8 (PMC12964632; doi:10.1186/s12870-026-08249-8)
Supplement: Supplementary file 1 — Supplementary Material 1. [file 12870_2026_8249_MOESM1_ESM.zip › Supplementary tables 2.docx]

|  | Differential hosts |  |  | *Yr* single-gene |  |
| --- | --- | --- | --- | --- | --- |
| No. | Name | *Yr* gene | No. | Name | *Yr* gene |
| 1 | Trigo-Eureka | *Yr6* | 1 | AvSYr1NIL | *Yr1* |
| 2 | Fulhard | Unknown | 2 | AvSYr5NIL | *Yr5* |
| 3 | Lutescens 128 | Unknown | 3 | AvSYr6NIL | *Yr6* |
| 4 | Mentana | Unknown | 4 | AvSYr7NIL | *Yr7* |
| 5 | Virgilio | *YrVir1*, *YrVir2* | 5 | AvSYr8NIL | *Yr8* |
| 6 | Abbondanza | Unknown | 6 | AvSYr9NIL | *Yr9* |
| 7 | Early Premium | Unknown | 7 | AvSYr10NIL | *Yr10* |
| 8 | Funo | *YrA*,*+* | 8 | AvSYr15NIL | *Yr15* |
| 9 | Danish 1 | *Yr3* | 9 | AvSYr17NIL | *Yr17* |
| 10 | JubilejinaII | *YrJu1*, *YrJu2*, *YrJu3*, *YrJu4* | 10 | AvSYr24NIL | *Yr24* |
| 11 | Fengchan 3 | *Yr1* | 11 | AvSYr27NIL | *Yr27* |
| 12 | Lovrin 13 | *Yr9*,*+* | 12 | AvSYr32NIL | *Yr32* |
| 13 | Kangyin 655 | *Yr1*, *YrKy1*, *YrKy2* | 13 | AvS/IDO377s(F3-41-1) | *Yr43* |
| 14 | Suwon 11 | *YrSu* | 14 | AvS/Zak(1-1-35-line1) | *Yr44* |
| 15 | Zhong 4 | Unknown | 15 | AvSYrSPNIL | *YrSP* |
| 16 | Lovrin 10 | Yr9 | 16 | AvSYrTres1NIL | *YrTr1* |
| 17 | Hybrid 46 | *Yr3b*, *Yr4b* | 17 | AvS/Exp 1/1-1 line74 | *YrExp2* |
| 18 | *Triticum spelta* var. *Album* | *Yr5* | 18 | AvSYr1NIL | *Yr1* |
| 19 | Guinong 22 | *Yr10*，*Yr26* |  |  |  |

**Table 2:** Chinese differential lines used to identify races of *Puccinia striiformis* f. sp. *tritici* isolates collected from six provinces of China in 2024.
