# Supplementary material for: Virulence and pathotype variability for Puccinia striiformis f. sp. tritici across different geographical regions and epidemic zones of China
Source: BMC Plant Biol. 2026 Feb 5;26:439. doi: 10.1186/s12870-026-08249-8 (PMC12964632; doi:10.1186/s12870-026-08249-8)
Supplement: Supplementary file 1 — Supplementary Material 1. [file 12870_2026_8249_MOESM1_ESM.zip › Supplementary tables 3.docx]

|  |  | |  | **Frequency (%)** | | |  | | |
| --- | --- | --- | --- | --- | --- | --- | --- | --- | --- |
|  | **Country** | |  | **G1** | | | **G2** | | |
| **Virulence** | **N** | **Freq. (%)** | | **SX** | **GS** | **QH** | **HN** | **HB** | **JS** |
| *Yr1* | 150 | | 71.77 | **76.4** | 66.7 | 88.24 | 75 | 65.3 | 68 |
| *Yr5* | 0 | | 0 | **0** | 0 | 0 | 0 | 0 | 0 |
| *Yr6* | 170 | | 81.34 | **84.7** | 100.0 | 82.35 | 75 | 77.3 | 76 |
| *Yr7* | 158 | | 75.59 | **83.3** | 66.7 | 76.47 | 87.5 | 77.3 | 64 |
| *Yr8* | 109 | | 52.15 | **76.4** | 75.0 | 76.47 | 62.5 | 74.7 | 64 |
| *Yr9* | 147 | | 70.33 | 73.6 | 83.3 | 88.24 | 37.5 | 64.0 | 72 |
| *Yr10* | 77 | | 36.84 | 30.6 | 25.0 | 35.29 | 37.5 | 44.0 | 40 |
| *Yr15* | 0 | | 0 | 0 | 0 | 0 | 0 | 0 | 0 |
| *Yr17* | 79 | | 37.79 | 41.7 | 33.3 | 23.53 | 62.5 | 37.3 | 32 |
| *Yr24* | 41 | | 19.61 | 36.1 | 33.3 | 23.53 | 50 | 36.0 | 52 |
| *Yr27* | 70 | | 33.49 | 36.1 | 33.3 | 35.29 | 50 | 26.7 | 40 |
| *Yr32* | 101 | | 48.32 | 51.4 | 25.0 | 64.71 | 12.5 | 48.0 | 52 |
| *Yr43* | 55 | | 26.31 | 22.2 | 25.0 | 29.41 | 50 | 28.0 | 24 |
| *Yr44* | 36 | | 17.22 | 22.2 | 25.0 | 17.65 | 12.5 | 13.3 | 12 |
| *Yrsp* | 141 | | 67.46 | 70.8 | 75.0 | 76.47 | 75 | 62.7 | 60 |
| *YrTr1* | 4 | | 1.91 | 4.2 | 0 | 0 | 0 | 1.3 | 0 |
| *YrExP2* | 128 | | 61.24 | 62.5 | 66.7 | 70.59 | 25 | 61.3 | 60 |
| *Yr76* | 122 | | 58.37 | 62.5 | 58.3 | 76.47 | 50 | 52.0 | 56 |

**Supplementary Table 3.** Virulence frequencies of *Puccinia striiformis* f. sp. *tritici* to 18 *Yr* genes countrywide and in various provinces of China
